# Supplementary material for: The Clinical Features of In-Hospital Recurrence in Acute Ischaemic Stroke Patients over Time: A Real-World Observation at a Single Center
Source: Brain Sci. 2022 Jan 18;12(2):123. doi: 10.3390/brainsci12020123 (PMC8869764; doi:10.3390/brainsci12020123)
Supplement: Supplementary file 1 [file brainsci-12-00123-s001.zip › brainsci-1515981-supplementary.pdf]

Table S1. The comparison of treatment and auxiliary examination between different stages

	Stage 1 (n=978)	Stage 2 (n=1047)	*p*-value
**Treatment**			
**IVT (%)**	87 (8.9)	52 (5.0)	0.001*
**EVT in 24h (%)**	7 (0.7)	12 (1.1)	0.315
**AntiPlt (%)**	939 (96.0)	1001 (95.6)	<0.001
None (%)	39 (4.0)	46 (4.4)	0.649
Mono (%)	551 (56.3)	290 (27.7)	<0.001*
Dual (%)	388 (39.7)	711 (67.9)	<0.001*
**Anticoagulant (%)**	35 (3.6)	92 (8.8)	<0.001*
**AntiHTN (%)**	474 (48.5)	666 (63.6)	<0.001*
**AntiDM (%)**	276 (28.2)	373 (35.6)	<0.001*
**Statin (%)**	890 (91.0)	1028 (98.2)	<0.001*
**Examination**			
**Holter (%)**	79 (8.1)	543 (51.9)	<0.001*
**UCG (%)**	909 (92.9)	995 (95.0)	0.048*
**CT (%)**	815 (83.3)	597 (57.0)	<0.001*
**MRI (%)**	940 (96.1)	981 (93.7)	0.014*
**CTA/MRA/DSA (%)**	868 (88.8)	998 (95.3)	<0.001*
**CVUS (%)**	895 (91.5)	976 (93.2)	0.148

* - significant difference (Bonferroni-adjusted P<0.0167 for AntiPlt). IVT, intravenous thrombolysis; EVT, endovascular treatment; AntiPlt, antiplatelet; AntiHTN, anti-hypertension agents; AntiDM, anti-diabetes agents; UCG, ultrasonic cardiogram; CTA, computed tomography angiography; MRA, magnetic resonance angiography; DSA, digital subtraction angiography; CVUS, carotid vessel ultrasound.

Table S2. Univariate analysis of the risk factors related to in-hospital recurrence in different periods

	Combined (n=2025)				Stage 1 (n=978)				Stage 2 (n=1047)			
**Factors**	**OR**	**95%CI**		**P value**	**OR**	**95%CI**		**P value**	**OR**	**95%CI**		***p*-value**
**Gender**	1.227	0.756	1.991	0.407	1.384	0.721	2.656	0.329	1.065	0.516	2.198	0.864
**Age**	1.013	0.109	0.997	1.030	1.016	0.995	1.038	0.114	1.010	0.985	1.035	0.451
**Previous IS**	1.005	0.618	1.632	0.985	0.740	0.377	1.452	0.382	1.419	0.701	2.873	0.330
**Previous TIA**	1.883	1.004	3.531	0.049*	2.469	1.116	5.460	0.026*	1.324	0.460	3.811	1.324
**Atrial fibrillation**	1.822	1.029	3.225	0.040*	1.403	0.617	3.191	0.420	2.452	1.101	5.460	0.028
**Hypertension**	1.134	0.725	1.773	0.582	1.127	0.630	2.017	0.687	1.177	0.584	2.372	0.649
**Diabetes**	1.863	1.245	2.787	0.002*	1.722	1.010	2.937	0.046*	2.184	1.170	4.077	0.014
**Paresis/ataxia**	1.134	0.682	1.889	0.626	1.235	0.675	2.260	0.494	1.376	0.491	3.860	0.544
**Aphasia**	0.879	0.539	1.421	0.591	0.462	0.241	0.885	0.020	1.980	0.952	4.119	0.068
**Coma**	2.005	0.941	4.272	0.071	0.000	-	-	0.998	4.312	1.901	9.783	<0.001*
**NIHSS score**	1.061	1.022	1.101	0.002*	0.992	0.921	1.069	0.843	1.098	1.051	1.146	<0.001*
**TOAST**												
**AA**	1.522	0.880	2.631	0.133	2.365	1.126	4.969	0.023$^{\#}$	0.913	0.404	2.063	0.828
**CE**	1.450	0.663	3.168	0.352	1.865	0.642	5.418	0.252	1.078	0.341	3.408	0.898
**SV**	0.039	0.005	0.298	0.002*	-	-	-	0.995	0.061	0.008	0.496	0.009*
**OD**	3.171	1.098	9.160	0.033*$^{\#}$	4.059	1.004	16.411	0.049$^{\#}$	2.300	0.447	11.840	0.319
**UD**	1.000				1.000				1.000			
**Pulmonary or urinary infection**	5.014	3.153	7.974	<0.001*	2.610	1.265	5.388	0.009*	9.050	4.776	17.149	<0.001*
**AntiPlt**	0.925	0.647	1.323	0.671	1.294	0.798	2.097	0.296	0.883	0.522	1.491	0.883
**DAPT**	1.031	0.689	1.545	0.881	1.452	0.853	2.471	0.169	0.845	0.443	1.610	0.608
**Anticoagulant**	2.374	1.288	4.381	0.006*	2.124	0.724	6.236	0.170	3.030	1.402	6.547	0.005*
**AntiHTN**	0.833	0.557	1.246	0.375	0.922	0.541	1.569	0.764	0.835	0.445	1.567	0.575
**AntiDM**	1.505	0.999	2.268	0.051	1.480	0.850	2.577	0.166	1.679	0.904	3.119	0.101
**Statin**	1.343	0.481	3.724	0.571	1.866	0.572	6.093	0.301	0.705	0.092	5.430	0.738
**IVT**	0.829	0.391	1.760	0.626	0.746	0.264	2.112	0.581	0.855	0.291	2.515	0.776
**hsCRP**	1.015	1.005	1.024	0.004*	1.004	0.989	1.020	0.586	1.029	1.014	1.044	<0.001*
**FBG**	1.078	1.006	1.157	0.034*	1.085	0.985	1.194	0.098	1.094	0.989	1.211	0.081
**Glucose abnormality**	2.761	1.664	4.582	<0.001*	4.046	2.111	7.757	<0.001*	1.768	0.766	4.085	0.182
**HbA1C**	1.123	1.103	1.245	0.028*	1.161	1.022	1.319	0.022*	1.088	0.919	1.288	0.329
**averageBP**	1.011	0.998	1.025	0.092	1.020	1.003	1.038	0.024*	1.004	0.984	1.024	0.714
**BPSD**	1.026	0.997	1.056	0.078	1.032	0.995	1.071	0.092	1.024	0.978	1.071	0.311

* - significant difference after Bonferroni correction ($p<0.0125$). $^{\#}$-significant difference before correction. IS, ischaemic stroke; TIA, transient ischaemic attack; NIHSS, the National Institutes of Health Stroke Scale; TOAST, the Trial of Org 10172 in Acute Stroke Treatment; AA, artery atherosclerosis; CE, cardio-aortic embolism; SV, small vessel disease; OD, other determined causes; UD, undetermined causes; AntiPlt, antiplatelet; DAPT, dual antiplatelet therapy; AntiHTN, anit-hypertension agents; AntiDM, anti-diabetes agents; IVT, intravenous thrombolysis; hsCRP, hypersensitive C-reactive protein; FBG, fasting blood glucose; HbA1C, glycosylated haemoglobin; BP, blood pressure; BPSD, the standard deviation of blood pressure, OR, odds ratio; CI, confidence interval.

Table S3. Univariate analysis of the risk factors related to in-hospital recurrence in large-artery atherosclerosis patients

	**Combined (n=946)**				**Stage 1 (n=429)**				**Stage 2 (n=517)**			
**Factors**	**OR**	**95%CI**		**P value**	**OR**	**95%CI**		**P value**	**OR**	**95%CI**		***p*-value**
**Gender**	0.931	0.519	1.670	0.811	1.038	0.490	2.200	0.922	0.874	0.342	2.233	0.778
**Age**	1.004	0.983	1.026	0.704	1.019	0.990	1.048	0.204	0.986	0.955	1.019	0.408
**Previous IS**	1.105	0.629	1.939	0.728	0.890	0.421	1.883	0.760	1.421	0.602	3.352	0.423
**Previous TIA**	1.628	0.802	3.305	0.178	2.191	0.850	5.646	0.104	1.357	0.452	4.078	0.586
**Atrial fibrillation**	2.260	0.495	10.318	0.292	1.637	0.192	13.946	0.652	3.233	0.375	27.892	0.286
**Hypertension**	1.094	0.624	1.917	0.754	1.134	0.548	2.346	0.735	1.072	0.441	2.606	0.878
**Diabetes**	1.942	1.171	3.222	0.010*	1.549	0.806	2.974	0.189	2.748	1.201	6.289	0.017*
**Paresis/ataxia**	0.872	0.464	1.638	0.669	1.066	0.503	2.258	0.867	0.928	0.269	3.194	0.905
**Aphasia**	1.073	0.605	1.903	0.809	0.660	0.320	1.361	0.260	1.718	0.667	4.422	0.262
**Coma**	2.344	0.950	5.784	0.064	-	-	-	-	5.365	1.985	14.499	0.001*
**NIHSS score**	1.051	0.998	1.107	0.057	0.949	0.858	1.049	0.302	1.114	1.048	1.184	0.001*
**Pulmonary or urinary infection**	4.080	2.200	7.568	<0.001*	1.995	0.721	5.520	0.184	8.742	3.753	20.362	<0.001*
**AntiPlt**	1.129	0.706	1.807	0.612	1.115	0.605	2.053	0.727	1.900	0.743	4.856	0.180
**DAPT**	1.190	0.711	1.991	0.508	1.288	0.672	2.471	0.446	1.865	0.690	5.040	0.219
**Anticoagulant**	4.161	1.485	11.660	0.007*	3.360	0.655	17.227	0.146	5.692	1.485	21.811	0.011*
**AntiHTN**	0.896	0.541	1.483	0.669	1.254	0.652	2.411	0.497	0.697	0.313	1.550	0.376
**AntiDM**	1.491	0.902	2.463	0.119	1.226	0.630	2.387	0.548	2.142	0.963	4.761	0.062
**Statin**	2.612	0.352	19.390	0.348	2.793	0.369	21.151	0.320	-	-	-	-
**IVT**	0.928	0.326	2.642	0.889	0.847	0.248	2.891	0.847	0.809	0.105	6.233	0.839
**hsCRP**	1.013	1.001	1.025	0.029*	0.998	0.978	1.019	0.827	1.043	1.021	1.066	<0.001*
**FBG**	1.075	0.987	1.171	0.095	1.057	0.934	1.195	0.381	1.118	0.992	1.259	0.067*
**Glucose abnormality**	2.751	1.524	4.964	0.001*	3.541	1.676	7.483	0.001*	1.828	0.663	5.041	0.244
**HbA1C**	1.075	0.945	1.222	0.271	1.104	0.945	1.290	0.211	1.047	0.842	1.300	0.680
**averageBP**	1.011	0.995	1.028	0.175	1.011	0.990	1.032	0.314	1.017	0.990	1.044	0.229
**BPSD**	1.006	0.965	1.049	0.771	1.008	0.958	1.059	0.769	1.014	0.949	1.084	0.680

*-significant difference. IS, ischaemic stroke; TIA, transient ischaemic attack; NIHSS, the National Institutes of Health Stroke Scale; AntiPlt, antiplatelet; DAPT, dual antiplatelet therapy; Anti HTN, anit-hypertension agents; AntiDM, anti-diabetes agents; IVT, intravenous thrombolysis; hsCRP, hypersensitive C-reactive protein; FBG, fasting blood glucose; HbA1C, glycosylated haemoglobin; BP, blood pressure; BPSD, the standard deviation of blood pressure, OR, odds ratio; CI, confidence interval.

Table S4. Univariate analysis of the risk factors related to in-hospital recurrence in minor stroke patients

	Combined (n=1336)				Stage 1 (n=635)				Stage 2 (n=701)			
**Factors**	**OR**	**95%CI**		**P value**	**OR**	**95%CI**		**P value**	**OR**	**95%CI**		***p*-value**
**Gender**	1.472	0.735	2.948	0.275	1.553	0.670	3.599	0.305	1.504	0.432	5.230	0.521
**Age**	1.011	0.990	1.032	0.322	1.017	0.991	1.044	0.202	0.996	0.962	1.032	0.821
**Previous IS**	0.951	0.486	1.862	0.883	0.724	0.312	1.679	0.451	1.315	0.429	4.034	0.632
**Previous TIA**	2.455	1.205	5.003	0.013	3.062	1.271	7.378	0.013	1.909	0.541	6.735	0.315
**Atrial fibrillation**	1.807	0.834	3.915	0.134	1.409	0.479	4.145	0.533	2.764	0.889	8.595	0.079
**Hypertension**	1.000	0.565	1.770	0.999	0.912	0.456	1.821	0.793	1.263	0.449	3.552	0.658
**Diabetes**	2.059	1.210	3.506	0.008*	2.062	1.067	3.983	0.031*	2.141	0.858	5.342	0.103
**Paresis/ataxia**	0.768	0.437	1.351	0.360	1.017	0.520	1.988	0.962	0.777	0.253	2.382	0.658
**Aphasia**	0.563	0.239	1.329	0.190	0.417	0.160	1.088	0.074	0.556	0.073	4.232	0.570
**Coma**	1.505	0.195	11.594	0.695	-	-	-	-	3.102	0.383	25.154	0.289
**TOAST**												
**AA**	1.467	0.735	2.926	0.277	2.631	1.056	6.555	0.038#	0.686	0.232	2.023	0.494
**CE**	1.622	0.610	4.315	0.332	2.211	0.595	8.216	0.236	1.152	0.264	5.018	0.851
**SV**	0.000	0.000	-	0.993	0.000	0.000	-	0.995	0.000	0.000	-	0.995
**OD**	1.917	0.399	9.205	0.416	4.533	0.808	25.427	0.086	0.000	0.000	-	0.999
**UD**	1.000				1.000				1.000			
**Pulmonary or urinary infection**	4.477	2.003	10.008	<0.001*	2.224	0.741	6.674	0.154	11.858	3.515	39.999	<0.001*
**AntiPlt**	0.784	0.504	1.222	0.283	1.167	0.640	2.128	0.614	0.663	0.326	1.349	0.257
**DAPT**	0.871	0.512	1.482	0.610	1.457	0.754	2.812	0.262	0.677	0.262	1.744	0.419
**Anticoagulant**	3.560	1.673	7.573	0.001*	2.924	0.818	10.454	0.099	6.236	2.267	17.150	<0.001*
**AntiHTN**	0.891	0.523	1.518	0.671	1.085	0.563	2.093	0.807	0.806	0.320	2.030	0.647
**AntiDM**	1.527	0.884	2.638	0.129	1.876	0.954	3.688	0.068	1.233	0.479	3.176	0.664
**Statin**	1.713	0.410	7.158	0.461	2.124	0.499	9.032	0.308	-	-	-	-
**IVT**	1.766	0.683	4.568	0.241	1.511	0.441	5.177	0.511	2.242	0.498	10.101	0.293
**hsCRP**	1.022	1.007	1.038	0.005*	1.015	0.994	1.036	0.163	1.031	1.007	1.055	0.010*
**FBG**	1.094	1.000	1.197	0.051	1.101	0.988	1.228	0.083	1.112	0.956	1.293	0.169
**Glucose abnormality**	3.244	1.661	6.337	0.001*	4.094	1.819	9.211	0.001*	2.134	0.603	7.550	0.240
**HbA1C**	1.146	1.003	1.310	0.046*	1.179	1.015	1.31	0.032*	1.073	0.822	1.400	0.603
**averageBP**	1.016	0.998	1.034	0.074	1.032	1.009	1.054	0.006*	0.997	0.967	1.028	0.870
**BPSD**	1.022	0.980	1.065	0.318	1.039	0.993	1.088	0.100	0.987	0.899	1.083	0.784

* - significant difference after Bonferroni correction ($p<0.0125$). #-significant difference before correction. IS, ischaemic stroke; TIA, transient ischaemic attack; NIHSS, the National Institutes of Health Stroke Scale; TOAST, the Trial of Org 10172 in Acute Stroke Treatment; AA, artery atherosclerosis; CE, cardio-aortic embolism; SV, small vessel disease; OD, other determined causes; UD, undetermined causes; AntiPlt, antiplatelet; DAPT, dual antiplatelet therapy; AntiHTN, anit-hypertension agents; AntiDM, anti-diabetes agents; IVT, intravenous thrombolysis; hsCRP, hypersensitive C-reactive protein; FBG, fasting blood glucose; HbA1C, glycosylated haemoglobin; BP, blood pressure; BPSD, the standard deviation of blood pressure, OR, odds ratio; CI, confidence interval.

Table S5. Multivariate analysis of the determinants associated with the antiplatelet regimen choice in noncardiac stroke patients.

	Conserved				Positive			
**Factor**	**OR**	**95% CI**		**P-Value**	**OR**	**95% CI**		***p*-Value**
**Age (≥75 years)**	1.739	1.348	2.243	<0.001*	0.805	0.578	1.121	0.198
**Previous IS**	1.063	0.812	1.391	0.657	1.306	0.970	1.758	0.078
**Diabetes**	0.997	0.690	1.440	0.988	0.853	0.552	1.319	0.475
**Paresis/ataxia**	0.554	0.430	0.714	<0.001*	3.317	2.117	5.198	<0.001*
**Aphasia**	0.764	0.581	1.005	0.054	1.471	1.101	1.967	0.009*
**Coma**	0.282	0.102	0.781	0.015*	2.210	1.244	3.925	0.007*
**AA**	0.760	0.572	1.009	0.057	1.050	0.754	1.463	0.772
**SV**	1.254	0.926	1.698	0.143	0.755	0.508	1.123	0.165
**AntiDM**	0.709	0.482	1.042	0.080	1.333	0.857	2.073	0.203
**Statin**	0.177	0.104	0.303	<0.001*	1.452	0.528	3.992	0.470

* - significant difference after the Bonferroni correction (p<0.025). IS, ischaemic stroke; AA, artery atherosclerosis; SV, small vessel disease AntiDM, anti-diabetes agents, OR, odds ratio; CI, confidence interval..

Table S6. Factors associated with the incidence of haemorrhagic events in Stage 2

	**No-Haemorrhage (n=1019)**	**Haemorrhage (n=28)**	***p*-value**
AntiPlt (%)	979 (96.1)	22 (78.6)	0.001*
DAPT(%)	704 (69.1)	7 (25.0)	<0.001*
TOAST			
AA (%)	510 (50.0)	7 (25.0)	0.012#
CE (%)	72 (7.1)	13 (46.4)	<0.001*
NIHSS score			
≤3 scores (%)	690 (67.8)	10 (35.7)	<0.001*
≤5 scores (%)	849 (83.4)	14 (50.0)	<0.001*

* - significant difference after Bonferroni correction (p<0.01). #-significant difference before correction AntiPlt, antiplatelet; DAPT, dual antiplatelet therapy; TOAST, the Trial of Org 10172 in Acute Stroke Treatment; AA, artery atherosclerosis; CE, cardio-aortic embolism; NIHSS, the National Institutes of Health Stroke Scale.
